# Laennec on Diseases of the Chest

**Published:** 1829

**Authors:** 


### REVIEWS AND BIBLIOGRAPHICAL NOTICES.

---

ART. VIII.—*Lænnec on Diseases of the Chest.* Philadelphia, 1823.

*Collin on the Stethoscope.* Boston, 1829.

Review concluded.—Diseases of the Heart.

We resume the STETHOSCOPE, and propose to inquire into its powers in the diagnosis of maladies of the HEART.

There are few diseases which excite a more withering anxiety in the patient than those of the heart. This seems to arise, principally, from three causes. First—The sympathies and functional dependencies of the other organs upon this great central engine of the circulatory system. Secondly—The known irremediable character of most of its organic læsions. Thirdly—The obscurity in which the just conception of the patient, involves the particular nature of these structural derangements. Every physician, therefore, should consider himself called upon to study them in all the modes which accident, clinical observation and inventive genius have devised for their illustration. Much of the darkness which once hung over them, has been dissipated within the last 20 or 30 years; but this dissipation is not to the amaurotic, the purblind or the comatose—the sleepers and dreamers of the profession—who open their eyes, only when they hear something like the metallic tinkling (*tintement metallique*) of Lænnec; and then direct them upon the folios of their ledgers, instead of the pages of that distinguished physician and the writers of his school. No, to all such, the streams of light which have been let into the chambers of the heart disclose nothing, and the sounds which its troubled movements send forth, are but noise: —they practice no *auscultation*, but that which informs them when an applicant has given *pulsation* to their knockers; and listen attentively to no instruction, but that which guides them to the patient's house.

To those, however, who look more to the means and less to the end—the final end—of their labors; who think nothing in the study of the profession below their notice, nor above their comprehension—although it may really be; who augment their efforts, when the difficulties augment; who delight to explore the hidden recesses of the system, because they are hidden; and feel 'conscience-bound' to bestow great attention, on whatever deeply interests their patients;—to this most respectable minority, the writings of Corvisart, and Allen Burns, and Lænnec, and Collin, and Johnson—with their collaborators—present in relation to the heart, enough to give precision to their diagnostic inquiries, and a character of science, if not of success, to their prescriptions.

In every individual belonging to the higher ranges of the animal kingdom, there are two organs, which exert on all the rest a sustaining influence so direct and immediate, that to wound them, in general, occasions speedy death. These are the *medulla oblongata* and the *heart*, both of which are, therefore, placed far beneath the surface, and surrounded by bony parieties. In thus guarding them from external violence, Nature has so disposed of them, as to throw many difficulties in the way of our clinical investigations. Our object is to inquire by what means those which relate to the *heart* are to be overcome.

In common with other organs, the HEART and its membranes are liable to inflammation and the various consequences of that morbid state. But in addition to these, its muscularity, cavernous structure, and mechanical functions render it obnoxious to numerous læsions, which are seldom met with in other parts of the body. Until the time of Baron Covisart, the diagnosis and pathology of these maladies, were but little understood; and even the last results of his inquiries left numerous *desiderata*. Another means of diagnosis was, indeed, required; and this has been supplied by Lænnec in the invention of the STETHOSCOPE.

After an observation of the symptoms, the chief reliance of Corvisant was upon Percussion;—a method not unworthy of adoption; but still more inefficient in the diagnosis of cardiac, than of pulmonary affections. It is possible, however, without the auscultation to establish the existence of *a* disease of the heart. Nothing in general is easier; but to determine its specific nature is the task; and without the aid of the Stethoscope we venture to pronounce that this in general is impracticable. The Cylinder then is an instrument of particular rather than general diagnosis, and therein consists its value.

But let it never be supposed that it will afford either particular or general information to any and every one. The fact is far different. Practice and experience on the movements of the heart, *in health*, are indispensable to a correct estimate, by the instrument, of the condition of that organ in disease. The physiological movements must be well understood, before the pathological can be appreciated. Hence we must begin with the former and end with the latter; and we must not limit our experiments to a single subject; but by trials on those of all ages, of both sexes, and of every temperament and condition, become familiar with all the modifications of sound and impulse, which the healthy organ is wont to impress upon us. In the course of these observations, we shall be struck with the fact, that the heart, in different persons, has quite as great a variety of movements, as, in figurative language, it exhibits of moral affections; and many of these might be mistaken for diseased actions, to the discredit of auscultation and the shame of the profession.

On the physiological movements of the heart, as indicated to us by the Cylinder, Lænnec is ample and original. His treatise should be read by all who aspire to the use of that instrument. Collin, with a happy faculty for condensation, has given us a brief and lucid view of this part of the subject; which we shall present our readers, to enable them by the practice of physiological, to become well prepared for pathological auscultation.

### Natural Phenomena furnished by the Heart.

These may be divided into four classes, and comprehend; 1st, The extent of the heart's pulsations; 2d, The shock which they communicate; 3d, The noise which accompanies them; 4th, Their Rhythm.

1st. *Extent of the palpitation of the heart.* In a healthy man of a moderate degree of plumpness, whose heart is of regular proportions, the beating of the heart can only be felt in the precordial region, that is to say, the space comprised between the fifth and seventh sternal ribs on the left side, and under the lower part of the sternum. The motions of the left cavities are felt in the first point, those of the right cavities in the second. When the sternum is short, the pulsations are still sensible in the epigastrium.

In very fat persons, the pulsations of whose heart cannot be felt by the hand, the space in which they can be detected by the aid of the cylinder, is sometimes limited to a surface of about a square inch. Very thin persons, with narrow chests, offer the opposite disposition; the pulsations of the heart have more extent, and may be felt in the third or even three-fourths of the inferior part of the sternum, sometimes under the whole of this bone, in the left superior part of the chest as far as the clavicle, and even beneath the right clavicle.—When the extent of the pulsation is limited to the points just mentioned, in persons such as we have pointed out, and they are less sensible under the clavicles than in the precordial region, the heart is still in good proportion.

2d. *The Shock.* I understand by shock the sensation of impulse or percussion against the ear of the observer, arising from the pulsations of the heart. It is distinct with the cylinder when the hand applied upon the region of the heart can perceive nothing. This shock is very considerable in a healthy man, particularly if tolerably plump. It is felt most in the precordial region and lower half of the sternum, and always with most force between the cartilages of the fifth and sixth ribs, the part corresponding to the point of the heart. Its force varies greatly, according to the constitution of the individual; it is therefore difficult to refer it to a uniform type.—Practice teaches us to distinguish whether it be more or less intense than it ought to be; it should be rather less for the right ventricle than for the left.

3d. *The Noise.* The alternate contractions of the different parts of the heart return a sound insensible in the healthy state, but easily perceivable by the cylinder, however small may be the strength or volume of the organ.

In the natural state this sound is double, and each pulsation of the heart corresponds to two successive sounds.

One clear, abrupt, analogous to the sound of the clapper of the bellows, corresponds to the systole of the auricles; the other more flat and prolonged, coincides with arterial pulsation, as well as with the sensation of the shock mentioned in the preceding article; it is produced by the contraction of the ventricles.

The noise of the right cavities is heard at the lower part of the sternum; that of the left cavities between the cartilages of the ribs. It is always stronger in the precordial region than in the other points of the chest, where it may become developed in persons, who, though otherwise healthy, have a heart with very thin walls. We observe also in them, that the sound of the auricles is more sonorous under the clavicles than that of the ventricles, which does not exist in the precordial region. In persons in whom the pleura and anterior border of the lungs is prolonged before the pericardium, the noise of the auricle is duller and more obtuse than that of the ventricles, without ceasing to be distinct. That depends without doubt on its being masked by the murmur of respiration, or by that produced by the air pressed out from this portion of the lung, by the heart's pulsation.

4th. *Rhythm.* We understand by the term Rhythm, the order of the contractions of the different parts of the heart, as they are heard by the cylinder, their respective duration, their succession, and general relation to each other. In a healthy man, whose heart is in a state favorable to the performance of all its functions, at the moment in which the artery strikes the finger, the ear applied upon the cylinder is gently raised by a movement of the heart, isochronous to that of the artery, and accompanied with rather a dull sound; it is the contraction of the ventricle. Immediately after, and without any interval, a shorter, louder sound announces the contraction of the auricle; no movement sensible to the ear accompanies this sound. An interval of repose succeeds to it. This interval, although short, is well marked. After it a new complete contraction of the heart is perceived.

The respective duration of the contraction of the auricles and ventricles appears determined with sufficient precision as follows. Of the total period taken up by a complete contraction and repose of the heart, a third to a fourth is occupied by the systole of the auricles; rather less than a fourth by the absolute repose; the rest by the contraction of the ventricles. These relations exist, whatever be the swiftness or slowness, the frequency or rarity of the movements, when the organ is healthy and well proportioned. pp. 33–37.

Having by repeated trials become familiar with the normal movements of the organ, the practitioner has acquired a standard of comparison for the abnormal, and may then commence their study. To advance prosperously in this, he should seize every occasion that may offer, and with industry and perseverance, ascertain and record all the varieties of morbid impulse, sound, and rhythm which it may present; carefully connecting them with the other symptoms; and when opportunity may, finally offer, with the morbid anatomy. In this manner, and this only, can he hope to read the *secrets of the heart*, and become a good clinical auscultator.

On the elements of this branch of semeiology we shall again avail ourselves of the *expose* of Collin.

### Morbid Phenomena presented by the Heart.

These pathologic phenomena, like the natural ones, are referable; 1st, To the extent in which we hear the pulsations of the heart, by the aid of the cylinder; 2d, To the shock or impulse of the organ; 3d, To the nature or intensity of the noise caused by its contractions; 4th, To the rhythm, according to which its different parts contract.

1st, *Extent.* The extent of its pulsations may exceed their natural limits, or be confined to a very small surface. But before entering into any details on this subject, it is necessary to establish a distinction between the extent in which we hear the pulsations, and that in which they may be felt.

The increase of extent in which the pulsations become evident, is usually in the following order: 1st, The left side of the chest, from the axilla to the region corresponding to the stomach; 2d, The right side in the same extent; 3d, The left posterior part of the chest; 4th, Lastly, but rarely, the posterior part of the right side. The intensity of the sound is progressively less in the succession indicated.

The possibility of hearing the heart in these different regions, always indicates a state of weakness of the organ, a small degree of thickness of its walls, particularly of the ventricles; the passive dilatation of some of its parts.

It may also depend on causes not connected with the heart, the action of which may be either permanent or temporary; such are the thinness or narrowness of the chest, hepatization of the lung, its compression by effused fluid, the existence of excavations with firm walls, pneumo-thorax, nervous agitation, an intense fever, palpitation, hemoptysis, and in general all the causes which increase the frequency of the pulse.

The diminution of the extent in which the heart's pulsations can be heard, usually announces a more or less marked thickening of its walls; it is not very often observed.

It is less rare to observe, that in the same circumstances, the pulsations of the heart seem to confine themselves so as to be felt in a smaller extent than in the sound state; on the contrary, when the heart is dilated, it strikes the sternum by a large surface. The two last observations are not always correct: I have met with numerous exceptions.

2d, *Impulse.* We have said, how many varieties the intensity of the shock of the heart in the healthy state offered to the ear, so it is very difficult to pronounce decidedly upon its increase or diminution, unless they are strongly marked, or do not exist at both sides at once, which is generally the case.

The increase of impulse offers numerous degrees, from a slight excess of force, to that violent shock which communicates a disagreeable stroke to the head of the observer, and raises the walls of the thorax strongly enough to be visible at a certain distance. It is almost always in direct proportions to the thickness of the ventricular walls, and in inverse proportion to the extent of the pulsations: it is then the pathognomic sign of hypertrophy of this organ.

Quick walking, running, ascending, nervous agitation, palpitation, fever, may produce it temporarily without any alteration of the heart; so we should not proceed to an examination till after a considerable repose of body and mind. Venesection lessens this impulse; we should judge badly of its degree after this operation.

The diminution of the impulse, though rarely, is sometimes as exaggerated as its increase; it depends at one time on the weakness of the organ, the small degree of thickness of its walls, and coincides with extended pulsations; at another, on extreme obstruction of respiration, on the difficulty of pulmonary circulation, and may then coexist with a well characterized hypertrophy. This diminution is often observed in the latter stages of the complaint; certain affections of the mind, fear, depressing passions, may also produce it.

3d, *Sound.* The sound of the contractions of the heart may become dull or more clear, more sonorous than in the natural state. It may give rise to sounds wholly new, of which not even the rudiments can be perceived in the healthy state of the organ.

The diminished intensity of the sound seems to depend on the softening of the tissue of the organ, or on an increase of the thickness of its walls; it is, with feebleness of impulse, the only sign furnished by the cylinder of the first of these affections, a disease rare and most usually mistaken.

The clearness, the sonorousness of the contractions is much more frequently observed; it is always met with in a heart having thin walls, and indicates this natural or pathological condition. This clear noise may proceed from the auricles or the ventricles. The time and place at which it is heard, indicate the part which gives rise to it; it always announces a thinness of the portion of the heart of which the contraction takes place.

As to the sounds which present themselves, not having any rudiment in the healthy state, we shall refer them to three heads. 1st, The bellows blast; 2d, The rasping noise; 3d, That which has some resemblance to the creaking of new leather.

1st, *The bellows blast,* tolerably well characterized by its name, may accompany the contraction of the different parts of the heart, or that of the arteries, either all at once or partially. It may be constant, or only return from time to time without any appreciable cause, on the slightest movement, or from the slightest emotion. The nervous, the hysterical, and hypochondriacal, those subject to any hemorrhage, present this phenomenon most frequently, without any alteration of the heart's structure, or any disturbance of its functions. It may also exist during disease of this organ in persons of none of the above dispositions.

At the opening of the dead bodies of those who have presented it in the highest degree, and in the most constant manner, in the heart and arteries, we do not meet any constant lesion to which it may be reasonably attributed.

M. Lænnec regards it as indicative of a simply spasmodic state of the circulating vessels, or of some part only of the sanguineous system. Many observations seem to prove this opinion:—1st, Its analogy to the noise produced by a forced muscular contraction, of which it is easy to assure ourselves. In fact, if we support the elbow upon a table, rest the ear forcibly upon the hand, and contract and relax the lower jaw alternately with energy, we hear a sound quite similar. 2d, The facility with which we cause it in some persons in a great number of arteries alternately, by resting lightly upon a point of the vessel, contracting its calibre, and thus offering to the course of the blood an obstacle which does not completely retard its progress. 3d, Its appearance before active hemorrhages in the vessels which carry the blood to the part through which the hemorrhage is about to take place. 4th, Its constant existence in cases of palpitation produced by anemia.

2d, *The noise of the rasp or file*, of which the denomination gives an exact idea, which it is impossible to mistake when once it has been heard, may, like the bellows blast, accompany the contraction of any of the parts of the heart; but it is not intermittent like that, its strength alone varies; once developed it ceases no more. M. Læennec regards it as a certain sign of contraction of the orifices of the heart by ossifications, vegetations, or other cause. The period of the contraction, and the place in which it takes place, indicate the orifice affected. Does not the possibility of producing a very similar noise in a person subject to the bellows blast, by pressing on an artery with a certain force, appear to announce that this is but a modification of the other, owing to a more marked state of spasm caused and maintained by an obstacle more difficult to overcome and always equally resisting?

3d, *The sound resembling the cracking of new leather* has only once presented itself to our observation; it was in a man who was carried off by a chronic pericarditis. The sound lasted during the first six days of the disease, and disappeared as soon as the local symptoms announced a considerable effusion into the pericardium.

Perhaps this noise may be a constant symptom of pericarditis before the occurrence of effusion into the serous envelope of the heart, a fugaceous symptom, when the disease terminates in a few days; of longer duration, when it is chronic.

4th. *Rhythm.* The changes in the rhythm of the heart's pulses are not very rare; but for want of sufficient observations, we have not been able to convert them into diagnostic signs.

They often accompany an hypertrophy, a dilatation of the heart, or the contraction of its orifices, during the whole duration of the complaint; they often manifested themselves also only in the latter stages of these affections; at other times these lesions produce death, without the rhythm of the pulsations having been irregular.

We shall consider the alterations of the rhythm; 1st, Relative to the respective duration of the contractions of the auricles and vetricles; 2d, Relative to their succession.

1st. The alterations of the rhythm rarely depend on the duration of the auricular contractions; they are usually owing to the increased length or the shortness of the ventricular contractions, and then it is the period of repose which is increased or diminished. The first of these alterations, that which consists in the prolongation of the ventricular contraction, is observed in hypertrophy, and is the more evident as the disease is more marked. The second, that in which there is greater rapidity of contraction, coincides with different states of the pulse, swiftness, rarity, and furnishes no data upon which we can found our diagnostic of diseases of the heart and lungs.

2d. These alterations, considered as they relate to the succession of the pulsations, have been sufficiently often observed; they are usually transitory, and rarely attend more than two or three complete contractions of the heart.

Thus sometimes the contraction of the auricle anticipates that of the ventricle; at others, that of the ventricle anticipates the auricular contraction; or again, a contraction of the ventricle is followed by many successive, rapid, as it were, convulsive contractions of the auricle, which, taken together, do not exceed the duration of an ordinary contraction.

The greater part of these anomalies do not produce any sensible change in the state of the pulse, and are not constantly met in any of the diseases of the heart.

In the midst of a series of contractions equal to each other, we often observe many shorter, more lively beats, after which the heart returns to its natural rhythm.

At other times, after a series of regular pulsations, the heart seems to stop, and remain for a very long time in a state of repose. These kinds of intermissions, observed in an adult, are always the sign of an affection of this organ.

In fine, in some more rare cases the swiftness and irregularity of the contractions are such, that it is impossible to analyze them. In this case we may pronounce with certainty that there exists a disease of the organ.

We see from this explanation of the pathologic phenomena furnished by the heart, that there are only two, impulse and sound, which become certain signs of lesion of different parts of this organ; that all the others drawn from the rhythm, bellows blast, and rasping sounds, &c. have not been sufficiently often observed to enable us to say what alterations they indicate. But I doubt not that attentive observation of those diseases, and their daily observation with the cylinder, may one day furnish the information now wanting, and very soon render the diagnosis of these affections as precise and easy as most of the other diseases of the thorax.—pp. 64–77.

### General Semeiology.

Before proceeding to inquire into the symptoms and auscultic phenomena which distinguish particular læsions of the heart, it may be well to recollect those which are in some degree common to this family of maladies. The most important are:—

1. Alterations in the force, fullness, frequency and rhythm of the contractions of the organ; increased by certain postures and especially by exercise—attended of course with a variable pulse.

2. Embarrassed respiration, without well developed cough, greatly increased by exercise.

3. An anxious and laborious expression of countenance, especially under great exertion.

4. Variations in the complexion, particularly of the face:

*a.* An unnatural paleness.

*b.* A purple or violet hue, sometimes only perceptible in the epithelia of the lips; or

*c.* A scarlet flush, which may appear under exercise, or arise and spread, rapidly and irregularly, over the visage of the patient, and disappear while you are yet conversing with him.

5. Headache, especially in the forepart, with great depression of spirits.

6. Wandering muscular pains about the chest.

7. Chylopoietic disorders, such as constipation, flatulence and biliary derangements.

8. In advanced stages, serous effusions into the cavities of the pericardium, pleura, peritoneum, and the cellular tissue of the extremities.

### Classification of Cardiac Maladies.

It would be absurd to suppose, that the Stethoscope is equally adapted to the diagnosis of every malady of the heart; but when about to indicate those concerning which it can be made to afford important information, it may be well, to place before us a systematic catalogue of the principal unnatural and morbid states of that organ. These may be grouped under the following heads:—

1. Malformations.
2. Muscular Læsions.
3. Degeneracies.

I.—*Malformations.*

1. The foramen ovale continuing open after birth.

2. The foramen ovale shut in the fœtus, driving all the blood into the pulmonary artery and changing the character of the pulmonary organ and circulation.—*Corvisart.*

3. The ductus arteriosus, continuing open.

4. The pulmonary artery preternaturally contracted or quite impervious, at the time of birth. A partial circulation through the lungs from the blood returning out of the aorta along the ductus arteriosus into the pulmonary artery.—*Dr. Hunter.*

5. Transposition of the aorta and pulmonary artery.

6. The aorta placed over the septum, and receiving blood at the same time from both ventricles.

7. A third ventricle placed between the others and receiving blood from both, while the two great arteries arise not from the right and left, but the middle cavity.—*Bell.*

8. Some of the valves wanting.

9. The whole heart preternaturally large or preternaturally small.

II.—*Muscular Læsions.*

1. Hypertrophy or unnatural growth:
   *a.* Of the left ventricle.
   *b.* Of the right.
2. Dilatation of the ventricles:
   *a.* Of the left.
   *b.* Of the right.
3. Hypertrophy with dilation:
   *a.* Of the left.
   *b.* Of the right.

4. Dilatation of the auricles with hypertrophy.
5. Aneurism.

III.—*Degeneracies.*

1. Fatty or adipose changes:
   - *a.* Deposits of fat around the heart.
   - *b.* Transformation of the muscular fibres into a fatty or adipocirous matter.
2. Bony or cartilaginous changes:
   - *a.* Of the valves,
   - *b.* Of the substance of the heart,
   - *c.* Of the coronary arteries.
   - *d.* Of the great vessels.
3. Ramollisement or softening of the heart.

To which may be added carditis, pericarditis, hydropericardium and spasmodic palpitations.

We shall now proceed to consider the diagnosis of particular maladies of the heart, passing by the congenital, and selecting from the remainder, such as are of most frequent occurrence, or best display the powers of the Stethoscope.

MUSCULAR LÆSIONS.

I.—*Simple Hypertrophy.*

*Diagnosis.* An excellent description of this disease was given by Allen Burns, of Glasgow, in 1809, in his work entitled "*Observation on some of the most frequent and important diseases of the Heart,*" but it remained for Lænnec, ten years afterwards, to give it the learned name of Hypertrophy, and to be regarded as the first who recognized its existence.

Mr. Burns observes, that in this disease "The balance between the heart and arteries is lost. By the great addition of muscular substance, the whole sanguiferous system is thrown into a deranged state of action." "There is confusion and struggling in the chest, inexpressible anxiety and inquietude in the action of the heart, with a weak, irregular, intermitting, fluttering, rapid, or corded pulse." The incessant and irregular action of the heart "Exhausts the bodily strength and overpowers the faculties of the mind, ruining every source of enjoyment and distressing the patient with never ceasing feelings of instantaneous dissolution."

To these diagnostics, Lænnec has added, that the patient seems to be constantly sensible of the action of the heart, but is not, as in some other cardiac læsions liable to spontaneous attacks of more violent palpitation.

The most accurate diagnosis is furnished, however, by auscultation. When we resort to the Stethoscope, we find great increase of impulse, with diminution of, or natural sound, and this impulse not extended over the chest, but limited, to the spots where the pulsations are usually felt. If it be equally strong, we may conclude that both ventricles are affected. As it may preponderate at the cartilages of the 5th and 6th ribs, or at the ensiforme cartilage, we decide that the left or right side is hypertrophied.

Sometimes the pulsations are irregular, intermittent; most commonly their rhythm experiences no other alteration than an increased duration of the ventricular contraction. Then that of the auricles takes place before that of the ventricles is finished; it is short, attended with little noise, and for that reason scarcely sensible.--Collin, p. 125.

The symptoms enumerated by Mr. Burns are those attendant on advanced stages of the malady. In its early period we can only rely on Auscultation.

Percussion rarely furnishes any results; however, in some cases of very severe hypertrophy, the sound has been observed to be obscure or flat in the præcordial region.—Collin, p.–124.

*Morbid Anatomy.*—Hypertrophia may exist in one or both ventricles, with or without a similar affection of the auricles. Most commonly the auricles are not affected, but occasionally they are so, while the ventricles are sound.

When affecting the left ventricle, I have seen its parietes more than an inch thick at the base, that is, double that of its sound state. Commonly, this morbid thickening diminishes insensibly from the base to the apex of the ventricle, where it is scarcely perceptible; sometimes, however, the apex partakes in the enlargement; as I have seen it from two to four lines thick, which is double or quadruple the natural size. The columnæ carneæ of the ventricle and of the valves acquire a proportionate enlargement. The septum between the two ventricles becomes also notably thickened in the disease of the left ventricle, (which fact seems to mark it as belonging to this rather than the other ventricle,) but never so much so as the other parts.

The muscular substance in these cases is of a degree of consistence sometimes double the natural, and is of a redder colour.—The cavity of the ventricle appears to have lost in capacity what its parietes have gained to thickness. Sometimes I have found this so small, in hearts twice the size of the first of the individual, as scarcely to be capable of containing an almond in its shell. The right ventricle, in such cases, is flattened along the septum, and does not extend to the apex of the heart. In extreme cases, it seems as if it were merely included within the parietes of the left ventricle.

In hypertrophia of the right ventricle the appearances are somewhat different. The thickening is here more uniform, and never so great as in the other: I have never found it greater than four or five lines. It is always a little greater in the vicinity of the tricuspid valves, and at the origin of the pulmonary artery. The columnæ carneæ are much enlarged, considerably more so, in proportion than those in the left, in disease of that side. Simple enlargement of the right ventricle, without dilatation, is much rarer than that of the left. When this disease affects both ventricles at the same time, the only difference from the description just given is, that each side assists to form the apex of the heart.—Lænnec, p. 166.

### II.—*Simple or Passive Dilatation of the Ventricles. Passive Aneurism* of Corvisart.

*Diagnosis.* As in the preceding species, the *general* signs of organic læsion will be present.

Mr. Burns observes, "in the cases of simple unmixed dilatation of the ventricles, which I have seen, the patients have complained of oppression in the chest, with a sense of occasional suffocation; the pulse has uniformly been full, soft and slow." "The heart beat sluggishly." He then cites a case in illustration, the principal symptoms of which were the following:—anxiety and oppression in the chest, accompanied with a constant disposition to syncope, upon making even trivial exertion. The paroxysms of breathlessness, continues he, were frequent, and the pulse fell from its usual standard, (70 to 80) at first to 18, then to 12, and, in the end, it was as low as 11 and 10 beats in a minute." It was full and soft. In this case, *all* the cavities were equally dilated; and the great arteries also, which undoubtedly contributed to the equanimity of the circulation.

Corvisart enumerates the following signs:

Lymphatic temperament; bland or timid disposition; antecedent and protracted grief, or debilitating disease of a chronic character. Visage rather pale and wan, or bloated and violet. The palpitations generally weak and dull. The sensation imparted to the hand of the observer when applied to the region of the heart, that of a large soft body, rising slowly against the ribs; and the slight impulse which it gives, apparently rendered weaker by the pressure of the hand. Pulse variable in frequency—generally weak and compressible.

To these signs it may be added from Lænnec, (p. 268) that in using the Cylinder, the impulse of the heart is found less than natural, but the sound emitted by the contraction of the ventricles is clear and loud; and not confined to a small space, but diffused in proportion to the extent of the dilatation.

When the left ventricle only is affected, the excessive sound is chiefly at the 5th cartilage, and the jugular veins are less distended. When the right ventricle alone is dilated, the greatest sound is at the lower end of the sternum; the jugulars are plethoric, but without pulsation; and the complexion is more violet.

The sound over the heart on Percussion is sometimes a little obscure.

*Morbid Anatomy.* Dilatation of the cavities of the heart is attended with diminished thickness of their parieties.

With these conditions, says Lænnec, there are commonly conjoined a notable degree of softening of the muscular substance, and a colour, either more violet, or paler, than natural. Sometimes the softness is so considerable, especially in the left ventricle, that the muscular substance can be destroyed by mere pressure between the fingers; and the parietes of the same ventricle may be so much diminished in thickness, as to be only two lines in the thickest point, and scarcely half a line at the apex, while the right ventricle is sometimes so completely extenuated, as to appear merely composed of a little fat and its investing membrane. The columnæ carneæ, particularly of the left ventricle, are more remote than in the natural condition of the part. The septum between the ventricles loses less of its thickness and of its consistence than the rest of the parietes.— Dilatation may be confined to one ventricle, (the other natural or hypertrophied) although it more commonly affects both at the same time. When one only is affected, the apex of it extends below the other, but not in so remarkable a degree as in the case of hypertrophia. The augmentation of the cavity seems to be more in its breadth than length. This is particularly observable when both the ventricles are dilated at the same time; as, at this time, the heart assumes a rounded shape, being nearly as wide at the apex as at the base.—Lænnec, p. 167.

III.—*Hypertrophy with Dilatation. Active Aneurism* of Corvisart.

1. *Of the left Ventricle. Diagnosis.* Sanguine temperament; robust constitution; the previous action of acute and violent causes.

Pulse variable, but generally hard and frequent.

Violent and struggling impulse of the heart, seeming to become greater from increased pressure of the hand.

The countenance injected, bloated and red.—*Corvisart.*

The Stethoscope reveals great sound and great impulse; more perceptible at the 5th, than the ensiform cartilage, but extending generally over the left side and even further.

The auricle is sonorous. The contractions of the ventricle are easily felt by the hand, even at a distance from the heart. The rhythm of the pulsations is rarely altered. Percussion causes an almost perfectly flat sound.—*Collin.*

The carotid, radial and other arteries are manifestly augmented in the force and volume of their pulsations.

2. *Of the right Ventricle. Diagnosis.* When this ventricle is affected with active aneurism, the pulse is less disturbed, but the face is more bloated and of a violet hue.—*Corvisart.*

The jugulars are distended and *pulsate.*—*Lancisi.*

The respiration is more embarrassed, and by exercise or internal stimulation, the dyspnœa is remarkably increased. Hæmoptysis, in various degrees, is a consequence that frequently recurs, especially under excitation of the heart.—*Burns.*

By auscultation, the great sound and impulse are found to be at the lower end of the sternum; but may be discovered in other parts according to the degree of dilatation.—*Lænnec.*

*Morbid Anatomy of the two Varieties.*—Dilatation of one ventricle is sometimes conjoined with hypertrophia of the other, but this is not so common as the complication in individual ventricles. I have met with the following varieties of this complication: 1st, Hypertrophia with dilatation of the left ventricle, and simple dilatation of the right; 2nd, Hypertrophia with dilatation of the left ventricle, and simple hypertrophia of the right; 3rd, Hypertrophia with dilatation of the right, and simple dilatation of the left; 4th, Hypertrophia of the right, with dilatation of the left: this last is the rarest. I do not remember to have met with hypertrophia of the left ventricle (with or without dilatation) complicated with dilatation of the right. I would even be disposed to consider such an union as impossible. Lænnec, p. 168.

IV.—*Dilatation of the Auricles, with Hypertrophy.*

*Diagnosis.* We know of no signs by which this rare læsion can be detected, except the absence of the symptoms of disease in the ventricles, and an increase of sound in the auricular contractions, may indicate it.

*Morbid Anatomy.*—I have never met with decided dilatation of the auricles without some thickening of their walls; and, on the other hand, I have never seen thickening of their walls without an augmentation of their capacity. I may here remark that it requires much experience to judge correctly of hypertrophia of the auricles, as, owing to their great natural thinness, a considerable increase (say double the natural thickness, and the increase is rarely so much) is not obvious to a person little accustomed to such examinations.

Dilatation of the auricles is an extremely rare disease, and it appears still more so compared with the frequency of the same affection of the ventricles. Sometimes we find in subjects affected with hypertrophia or dilatation of the ventricles, the auricles, also proportionably enlarged; it is, however, much more common to find these retaining their natural size even in cases where the ventricles are enormously enlarged. Sometimes also, but more rarely still, the auricles are dilated when the ventricles are of the natural size.—Lænnec, pp. 168–169.

V.—*Aneurisms.*

Time was, when all dilatations of the heart were grouped under the comprehensive term *Aneurism.* At present this designation is properly restricted, to such as bear the same anatomical relations to the parieties of the heart, as arterial aneurisms bear to the tunics of the vessels. Thus limited, the term embraces but a small number of cases, for true cardiac aneurisms, appear to be much rarer than simple dilatations and hypertrophies. Several well marked cases have, however, been recorded; and we are at liberty to suppose, that a still greater number have escaped observation, both before and after death. Of the recorded cases, the majority, if not the whole, have belonged to the left ventricle. When small, they seem to give rise to few symptoms, and none by which their existence can be established; and when large, their diagnosis is little less obscure. In a case reported by Cruveilheir, the predominant symptoms were those of a severe asthma, and nothing is more probable, than that many cases of that malady have depended on aneurisms or some other organic affection of the heart. When the aeurismal parieties give way, sudden death must of course be the consequence.

Aneurisms of the great arteries within the chest are of deep interest. In their symptoms and tendency, they may be compared with the most formidable of the læsions of the heart, which, however, they generally outstrip in their mortal career. They are apt to occasion instant and unexpected death, and have often not been supposed to exist, until detected in *post mortem* examinations.

*Diagnosis.* When the dilatation is not yet so great as to form an external tumour, the signs of this disease are liable to be confounded with those which in general terms indicate affections of the heart. In this stage of the disease, Corvisart relies chiefly on a hissing sound, which, however, manifests itself only when the tumour occupies the arch of the aorta and acts upon the trachea. A peculiar rushing, above the region of the heart, that organ being found in its usual place. A dull sound under percussion, of the upper and middle part of the thorax. Finally, the smallness and irregularity, of the pulse.

Lænnec regards these symptoms as equivocal, and expresses the opinion, that "in the present state of our knowledge there certainly exists no certain means of ascertaining the existence of this disease until it shows itself externally." His experience with the Stethoscope has not been sufficiently diversified, to afford any satisfactory results. In one case where the tumour had appeared externally, in which he was enabled to verify the diagnostic indications afforded by the cylinder, by dissection, "the pulsations of the tumour were perfectly isochronous with the pulse at the wrist; they gave at the same time a greater impulse and louder sound than the mere contraction of the ventricles; and the contraction of the auricles was not at all perceptible."

The intelligent Editor of the American edition of Collin observes, that—

In aneurism examined by the stethoscope, there is a peculiar sound, different from that of the heart, and different also from that of the great arteries in their natural condition (which it may be difficult if not impracticable to describe so as perfectly to convey the idea in words to one who has never observed the peculiarity upon the living body,) which is nevertheless characteristic of aneurismal dilatation. A person who has, in a single instance of well marked aneurism, carefully investigated and observed the phenomena perceptible to the sense of hearing by means of the stethoscope, will readily recognize this peculiarity of the sound. This is not confined to aneurism of the large internal arteries,—the Editor of the present edition having observed it in inguinal, femoral, popliteal, brachial aneurisms, as well as in those of the larger arteries within the chest and abdomen. In a case of varicose aneurism of the arm, produced by transfixing the vein and wounding the artery in bleeding, the sound produced, when examined by the stethoscope, might be compared to that of a circular saw acting upon wood, one small portion of the circle bearing in a less degree upon the wood than the remainder of the circle; but the diminution of the sound scarcely amounting to a perfect intermission.

In thoracic aneurisms of the great branches of the aorta, a comparison of the pulsations of the carotid and subclavian arteries among themselves, might, in connexion with the signs already enumerated, enable us to decide on the spot affected.

### Degeneracies.

### I.—*Adipose.*

*Diagnosis.* We know of no signs by which adipose degeneracies or envelopements of the heart can be identified. Depositions of fat might perhaps be inferred, from prevailing obesity, connected with disturbances of the circulation and the signs of softening of the heart.

Transformation of the muscular substance of the heart into fatty matter might be expected to be attended with the same signs as softening of the heart.

### II.—*Bony or Cartilaginous.*

1. *The Substance* of the heart is sometimes indurated to a cartilaginous state. Lænnec supposes this to be the effect of Hypertrophy. Sometimes it becomes partially osseous. Mr. Burns informs us, that Bordenave met with a case in which the whole heart was ossified. Mr. B. has described a case, (Margaret Henderson's,) in which both the ventricles, except about a cubic inch at the apex of the heart, were ossified, and as firm as the skull. Both auricles were thicker than usual, but healthy. Many cases of partial ossification are mentioned by authors.

The diagnostic symptoms have not yet been made out.

2. *Of the Valves.* Induration, ossification and excrescences of the valves of the left side, are much more common than of the right. These affections may bring on hypertrophy, or dilatation, and are often complicated with them.

*Diagnosis.*—Ossification of the mitral and sigmoid valves does not produce irregularity of the circulation, and cannot therefore be suspected from the state of the pulse, or by the application of the hand to the region of the heart, unless it is so considerable as materially to lessen the orifices of the left ventricle. In ossification of the mitral valve, in a middling degree, the sound which attends the contraction of the auricle becomes much more prolonged, more dull, and with something in its tone which reminds one of the rasping of a file on wood, and sometimes of a bellows smartly compressed.—This sound is well-marked when the *purring* is not perceptible to the hand, but it is much more distinct when this is perceptible, and is, indeed, proportional to its intensity.

The ossification of the sigmoid valves of the aorta is shewn by the existence of this sound during the contraction of the ventricle; but this does not exist in slight degrees of the affection, nor in a similar condition of the mitral.

In these cases, as in dilatation and hypertrophia, the alternate examination of the heart under the sternum and between the cartilages of the fifth and seventh ribs, as well as the state of the external jugulars, will always enable us to decide in which side of the heart the disease exists.—Lænnec, p. 272.

3. *Ossification of the Coronary Arteries;* attended with the symptoms which characterize and sometimes, constitute Angina pectoris, or Syncope anginosa.

*Diagnosis.* Sudden paroxysm of pectoral anxiety, with painful sense of suffocation, induced by exercise, going off after a total cessation of muscular effort. Pulse, in the attack, reduced in strength, irregular, and sometimes with the contractions of the heart entirely suspended, so as to produce or constitute syncope. Lancinating pains through the chest, from before backwards, in the pectoral and subscapulary muscles, and, often in the arms, especially, the left. Face bloodless and sweaty. Stomach oppressed with flatulence. After a time, the paroxysms returning when the patient is quiet or asleep. In the early stages the pulse, between the paroxysms, not particularly abnormal, but at last becoming habitually weak. Ultimately dropsical effusions, as in other cardiac læsions.

*Morbid Anatomy.* The coronary arteries generally ossified, and portions of ossification in other parts of the vascular system and the heart itself. That organ often loaded with fat, and sometimes partially transformed into adipose matter. The muscular fibres often relaxed and flabby. Sometimes softened.

### III.—*Ramollisement or softening of the Heart.*

*Diagnosis.*—We may consider this to exist when the heart returns a sound equally moderate, dull, and obtuse in its two contractions, and communicates little or no impulse. If the noise caused by the heart's contractions, although loud, is rather flat, and loses the clapping character usually attending dilatation, the softening is attended with that state; but if the sound of the contraction is so indistinct as to be almost no longer heard, it will be accompanied by hypertrophy.

Sometimes, however, in attacks of palpitation, a softened hypertrophied heart may produce brisk contractions, short, and like the blow of a hammer, but this effort is of short duration, and the organ very soon falls back into its habitual state.--Collin, p. 128.

Having in this Journal for October, 1828, reported a case of this disease, and given copious extracts from Lænnec on its diagnosis and morbid anatomy, we shall refer the reader to that number.

### Carditis and Pericarditis.

*Diagnosis.* In our October number for last year, we gave copious excerpts from Lænnec on these affections, which we shall not repeat.

As yet the value of the Stethoscope in the diagnosis of these inflammations, has been found very small. The manual of Collin may be regarded as presenting all that is known on this subject. The following are his principal remarks, which apply to inflammations of the pericardium, rather than the heart itself:—

M. Lænnec mentions the increased impulse and sound of the ventricular contractions, with their inequality and disproportion to the feebleness and smallness of the pulse, as signs of pericarditis. Perhaps the noise which I have compared to the creaking of leather, is a symptom of this affection; it is one of short duration, limited to a few hours, if the disease is acute, and quickly producing effusion, more durable when the progress is slower. The friction of the two lamina of the pericardium, deprived of the serous exhalation, by the inflammation, may explain the sound.

In the diagnosis of pericarditis the symptoms upon which we may, perhaps, place the greatest reliance, are a feeling of anguish in the region of the heart; disturbance and depression of the feelings and faculties of the mind; an irregular, small, intermitting and wiry pulse; occasional vomiting and sympathetic abdominal pain; a strong tendency to syncope on taking exercise; and the absence, under the use of the Stethoscope, of the signs of those cardiac læsions which have been described.

Both carditis and pericarditis are frequently metastatic diseases, and depend on a rheumatic or athritic condition of the system. Hence we may often derive aid in our diagnostic researches, by inquiring into the previous or most habitual maladies of the patient.

For the diagnosis and treatment of rheumatic affections of the heart and pericardium, the reader may consult Johnson on the Liver, which presents an interesting collection of cases, with many sound practical reflections.

When extensive adhesions of the pericardium to the diaphragm have taken place, a strong pulsation may become perceptible in the epigastrium.—*Burns.*

*Morbid Anatomy.*—Adhesions more or less general of the pericardium to the heart, the diaphragm, and the pleura pulmonum. Tubercular depositions of lymph. Thickening of the membrane. Serous and purulent effusions. Softening of the heart.

### Hydropericardium.

*Diagnosis.*—In genuine dropsy of the pericardium the breathing is generally less affected in all positions and circumstances of the body; and it is more of the quick, hurried, anxious, than of the slow laborious kind: in other respects there are some peculiarities. Though the recumbent posture be borne with less inconvenience, yet if the pericardium be much distended, and if the other cavities contain but little, or be entirely free from water, a sensation as if something were rolling in the breast, in the region of the heart, is perceived on turning from one side to the other, together with an increased difficulty of breathing, and a sense of weight, on attempting to lie on either, especially the right.

The erect position too is no less irksome, owing to the pressure and *bearing down pain* of the distended pericardium on the diaphragm, to relieve which the patient is obliged to sit up, or walk, with his body bent forward, or stooping. The greatest ease is in such cases experienced lying on the back with the shoulders elevated and the body inclined to the left side; this indeed will be found the easiest position in every stage of the disease.

The palpitation of the heart, though probably more constant, is less violent, and less distinctly felt, being of the weak fluttering kind; and this circumstance, added to the more remarkable variations observed in this respect on change of position, may serve to account in some degree for the great difference of opinion in medical writings in regard to its presence or absence.

The peculiarities of the pulse are still more striking; it is softer, more feeble, frequent, irregular and tremulous, without those occasional intermissions, now and then succeeded by a few regular and full strokes of the artery, mentioned as occurring in hydrothorax.—It will be obvious, however, that the palpitation and pulse will vary according to the stage of the disease or the quantity of water contained in the pericardium.

The peculiar sense of sinking, fainting, anxiety, or constriction, will be more constant, and referred more immediately to the seat of the heart.

With respect to the swelling of the ankles, defective secretion of urine, coldness of the extremities, and other deviations from the healthy state, mentioned as common to hydrothorax, experience does not warrant our reliance on any symptom which does not occur in the one as well as in the other.

A learned and ingenious medical friend, of whose judgment I entertain the highest opinion, has favored me with the following remarks and interesing case: "So far," he observes, "as my experience enables me to judge, under the guidance of my memory and written notes, I refer the diagnostic symptoms of the dropsy of the pericardium to *two* only, all besides being extremely uncertain, I mean *the feebleness and fluttering of the pulse*; and the ability of the patient to bear *the recumbent posture*.—McClean, p. 39.

Authors vary respecting the symptoms of this affection. Lancisi states the principal to be a sensation of an enormous weight in the region of the heart. Reimann and Saxonia assures us that the patient feels his heart swimming in water. Senac says he has seen the fluctuation of the fluid between the third, fourth and fifth ribs. M. Corvisart says he has perceived this fluctuation by the touch, and adds the following as marks of the affection:—sense of weight in the region of the heart; inferior resonance on percussion; pulsation of the heart irregular and obscure, and felt over a large space and with variable intensity in the same and different points of this space; pulse small, frequent and irregular; threatened suffocation on lying in the horizontal posture; frequent syncope, but rarely palpitation; œdema. To these symptoms I may apply the same remarks as to those of pericarditis: they may exist, in greater or less number, with or without hydro-pericardium. I am unable to say, from experience, how far, and in what respect, the cylinder will assist the diagnosis of this disease.—Lænnec, p. 275.

As to dropsy of the pericardium, the only symptoms which can lead us to suspect it, are a completely flat or fleshy sound in the precordial region, and tumultuous and obscure pulsations of the heart, sensible in a great extent, and for some moments more at one point than at others, and reaching the hand, as if a soft body were interposed.—Collin, p. 133.

PALPITATIONS AND SPASMODIC IRREGULARITIES OF THE HEART.

All the *organic* diseases of the heart which have been enumerated, are attended with habitual or occasional palpitations, intermissions, or other disturbances of its rhythm. They depend, first, on the embarrassment which a læsion of anatomical structure necessarily imposes on the heart; and secondly, on the morbid sensibility (contractility) to which those derangements give birth, or, at least, with which they are associated. But there are palpitations and other abnormal contractions of the organ, which arise exclusively from the latter of these two proximate causes, and in which the prognosis is very different from that which belongs to every case involving an organic affection, which is, in general, incurable.

*Diagnosis.* Now the problem is, to determine, in a case of palpitation or irregularity of action, whether it is caused by an organic læsion. In many cases this is a question of difficulty, and in all of deep interest to those afflicted.

When the enumerated signs of the different structural derangements, or, a majority of them, are manifest, we are not at liberty to indulge a doubt whether the palpitations or irregularities are the consequence of such derangement; but if those signs are uncertain, there is ground for hope that the palpitations are purely spasmodic; and when they are altogether absent, we may without much hesitation, assure the patient that his case is not dangerous. Prudence, however, dictates that we should form and express our opinions cautiously, as the most sagacious and experienced are liable to commit error in the diagnosis of these maladies.

Something may be inferred from the temperament of the patient. According to our own observations, those of the bilious and lymphatic and nervous temperament, are most liable to palpitation. A reference to age will likewise aid us. In males, palpitations are most common from 18 or 20 to 30 or 34—in females, about the periods of the establishment, and final cessation of the catamenial function. The sex should also be taken in account; as palpitations unconnected with organic læsion are more common in women than men.

A still greater reliance may be placed on the previous maladies of the patient or the isochronous disturbance in other organs. For the benefit of our junior readers, we shall enumerate the principal disordered states of the body in which we have met with those irregularities of the heart's action that are purely functional, that is, unaccompanied by inflammation or structural læsion.

1. *Irritation from Venesection.* Every observing practitioner must have witnessed the powerful influence of bloodletting, in the production of a state of morbid irritability of the heart and arteries. Hence, in the treatment of acute diseases, where something more than leeches, and the *medicine expectante*, compose the whole treatment, it is not uncommon to find our patients, in the decline, affected with palpitations and other 'nervous' disorders. The circumstances under which they occur will in general enable us to distinguish them from such as depend upon injuries of structure.

2. *Dyspepsia.* In this malady, the ordinary duration of which is twelve or fourteen years, or through the 4th and 5th climactericks, palpitations, intermissions, and irregularities of the movements of the heart are exceedingly common; and often occasion great alarm to the patient. The most common form is palpitation, which occurs oftener at night, in bed; than in the day, when the individual is occupied and the ordinary causes of mental excitement are acting upon him. But disturbances of the rhythm, of a graver and more suspicious character sometimes present themselves. Thus, we recollect a case in which the heart was affected with a strong, convulsive and tumultuary heaving, without any particular increase in the number of its pulsations. The patient was a physician between 30 and 40, who in the midst of dyspepsia had prosecuted his studies and practice until he was reduced to a skeleton, without having at any time had palpitations. In this condition he passed suddenly to a life of physical repose, and indulged himself in a full diet with moderate alcoholic stimulation. All his dyspeptic symptoms were forthwith signally mitigated; but in 10 or 12 weeks, he was awakened at midnight, by a violent, struggling and intermittent action of the heart, which continued for some hours. He lost blood and took cathartics, but they afforded little or no relief, and sometimes even seemed to augment the spasmodic action. It returned upon him at various times, especially at night, for 12 or 15 months, when it gradually abated, and at length ceased altogether. More than eight years have since elapsed without his having experienced an attack.

Purely spasmodic palpitation of the heart is pre-eminently a disease of students of medicine, most of whom in this country, are dyspeptic, at least during their attendance on medical lectures. Their habits are then more sedentary than before, they eat heartily, evacuate sparingly, abridge their hours of sleep, and study more diligently than usual. Thus they are in a course of physical and intellectual imbibition, and a state plethora, with morbid sensibility of the whole system is the consequence. In this condition, palpitations are exceedingly apt to supervene; and as most of this class of patients, have read of organic diseases of the heart, the apprehension that such may be their case, never fails to arise; and quite as certainly contributes to increase the spasms of the organ. When attached to the medical department of Transylvania University, we had numerous opportunities of witnessing all that is here described.

3. *Hypochondraism.* The state of the nervous system in this malady, at whatever period of life it may occur, is peculiarly favourable to the production of palpitations; while the morbid condition of the patient's feelings and faculties, never fail to convince him that his heart is incurably diseased.

4. *Chronic Hepatitis.* The morbid sensibility of the whole system, in this complaint is, in fact, one of its most striking pathological characters; especially after the depletion which is employed in the treatment of the preceding stage. The causes of this, are sufficiently obvious. In addition to the sympathies of the other organs with the liver, and the influence of depletion, which always heightens the susceptibility of the system, the elements of the bile are not sufficiently eliminated from the blood, nor the bowels adequately excited by their natural stimulus; and hence arises a state of perverted sensibility, not less complicated in its proximate

ætiology, than annoying in its effects. Among these, an enfeebled and palpitating condition of the central organ of the circulation, is one of the most constant, contributing greatly to the despondency of the unhappy, and too often unpitied, sufferer.

5. *Chlorosis.* In this disease, related in several pathologic traits to the last, disturbances of the functions of the chest, are extremely common. They present us, indeed, in different cases, with many well simulated cardiac and pulmonary diseases, all of which evanish under a method of treatment that would fail, entirely, to cure an organic, or any *original* affection, either of the heart or lungs.

6. *Menorrhagia.* Copious uterine hæmorrhages from whatever cause and whenever occurring, are peculiarly apt to be followed by palpitations, which from their very obvious origin, need never be confounded with those which depend on original diseases of the heart.

7. *Hysteria.* Of all the morbid states, which generate functional derangement of the heart, this is one of the most common. Let us hear Sydenham on this subject:—

"Sometimes it (*the hysteric passion*) seizes the vital parts, and causes so violent *a palpitation of the heart*, that the patient is persuaded, those about her must needs hear the heart strike against the ribs.—Slender and weakly women that seem consumptive, and girls that have the *green sickness*, are chiefly subject to this species."—Wallis' Ed. vol. II. p. 108.

By the way, Sydenham, whose work is an enduring evidence, that a man—*such a man as himself*—may be a skilful physician, without the modern aids of *physiology, morbid anatomy*, or *pharmaceutic chemistry*, this same Sydenham, now only read by the bibliothæcasts, has left us the best treatise (absurdly associated with another on the small pox) that is now extant, on the semeiology and therapeutics of hysteric diseases;—a treatise which we have re-perused with equal pleasure and profit, and which we would recommend to the study of all who have taste or ambition to look beyond the last number of the nearest medical journal. But to return to our proper object.

When the 'hysteric passion,' (we do not know a better phrase,) is fairly excited in the system, we need never expect the heart to escape; but we cannot often confound these palpitations with those originating in a læsion of its structure. The temperament, chilliness, previous attacks, transient diabetes, and fear of death, should the globus hystericus, even be absent, will sufficiently indicate to us, the situation of the patient; and, in general, justify that diagnosis which is most desirable. We have, however, more than once met with paroxysms of palpitation exhibiting various characteristics of hysteria, in females labouring under organic diseases of the heart; so that because hysterical symptoms happen to be present, we are by no means to decide positively that there is no original cardiac disease.

8. *Grief and Apprehension.* The influence of these and other depressing emotions, on the central organ of the circulation, is universally felt and acknowledged. There are temperaments and idiosyncracies, which resist their influence more than others, but on the whole it may be said, that the passions are among the most powerful of all the causes which disturb the equanimity of the heart, not only disordering its functions by the production of various spasmodic actions, but generating in it a number of serious organic læsions. Although, we may, therefore, clearly perceive that the palpitations of our patient originated in a previous affection of the sensorial functions, we should be aware, that after they have continued for a time, a structural derangement may have supervened.

Such are some of the disordered states of the nervous system which may carry disturbance in the movements of the heart, and excite the apprehension of organic disease. We might easily extend the catalogue, but shall leave the rest with our readers.

In relation to palpitations and irregularities from these different pathological states, we may remark, as serving still further to distinguish them from the abnormal movements in organic disease, that, 1. They are generally worse when the patient is warm and quiet in bed. 2. In the day, they rage more violently, when he is at rest than when in motion; but to this as to the preceding remark, there are exceptions; as we have seen palpitations, which, from the circumstances under which they occurred, seemed to be purely functional, that were, like those from organic disease, increased by exercise. 3. They are never habitual, while those from structural derangement in *most cases* are. 4. They are greater when the patient is idle or alone, and left to contemplate them, than when he is occupied or in company. 5. They are generally aggravated by venesection, which mitigates most of those from organic disease; but are signally relieved, and often, for the time being at least, entirely removed, by emetics and cathartics, or diffusible stimulants, narcotics and tonics, agents which afford but little relief when the structure is deranged.

The patient is more sensible of the palpitations and irregularities which arise from a spasmodic diathesis merely, than of those occasioned by organic disease, the reason of which, is to be found in the acute and unnatural sensibility attendant on the former condition.

Of what value is auscultion in the comparative diagnosis of organic and functional palpitations? This question has attracted the attention of Lænnec, and the results of his observations are set forth in one of the following paragraphs, which we extract from the Medico-Chirurgical Review, not having yet been able, to obtain the last writings of the French pathologist, from which they were taken:

"The purely nervous palpitations consist in an increase of the impulse, sound, and particularly of the frequency of the heart's pulsations. A feeling of internal agitation, particularly in the head and abdomen, always accompanies them; also a limpid watery condition of the urine. The duration of palpitations of this kind is very variable: they may be momentarily excited by mental emotion; while, at other times, they seem to originate without any obvious cause, and continue for several years, especially in young persons who are at the same time both nervous and plethoric. It is commonly imagined that such an habitual over-action of the heart as such palpitations imply, must at length give rise to hypertrophy of this organ. This is possible; but I must say that I have never seen any proof of the accuracy of this opinion. On the other hand, I am acquaited with individuals who have been habitually subject to affections of this kind, and who nevertheless exhibit no positive sign either of hypertrophy or dilatation."

"In nervous palpitation, the first impression conveyed by the stethoscope is that the heart is not enlarged. The sound, though clear, is not heard loudly over a great extent of chest, and the impulse, although appearing considerable at first, is really not great, as it never sensibly elevates the head of the observer. This last sign seems to me the most important and certain of any, when taken in conjunction with the frequency of the pulsations. These are always quicker than natural,—being, most frequently, from eighty-four to ninety-six in the minute. Nervous palpitations are rarely accompanied by any sign of determination of blood to the head or chest, except in old persons."

With these quotations we close our remarks on the signs by which we may know mere palpitations; and shall conclude our diagnostic inquiries, by a reference to the symptoms which characterize

### Rheumatism of the Heart.

When the rheumatic passion (to imitate the phraseology of Sydenham) enters the fibrous structure of the heart and pericardium, an immediate change in its mode of action ensues, but the abnormal movements are too various to be indicated by any single expression. Sometimes the number of pulsations is reduced far below, at others increased far above the healthy standard in the individual; in all cases their rhythm is overthrown, and in most the organ contracts with a violent, convulsive, and inflammatory energy; which is greatly increased by exercise. The patient experiences præcordial pain and anxiety; the general signs of fever are present; lastly, rheumatism of the joints has pre-existed, and commonly ceases as the cardiac symptoms are developed. An attention to other symptoms would seem to be unnecessary.

We have now finished what we at first proposed, the consideration of the Stethoscope in the diagnosis of maladies of the heart, and might here terminate our review. As we are at liberty to presume, however, that our readers, like ourselves, have got their minds turned towards the *treatment* of this important and interesting class of diseases, we shall venture to extend our article beyond the limits originally intended.

But before entering on their therapeutics, it will be proper to study them a little further; and in the first place, of the—

DISORDERS WHICH THEY OCCASION.

The researches of modern anatomists, and especially of Scarpa, have shown the absurdity of those who alleged that the heart has no nerves. It now appears that this organ is abundantly supplied, from a plexus which is made up of cephalic, spinal and ganglionic branches, but chiefly the last. It is thus connected, physiologically, with every other organ, and able to compel them to sympathise in its various affections. We might then, *a priori*, suppose, that in its morbid states, it readily excites disturbances in the functions of the other viscera, and such we find to be the fact. Of these the lungs, having with it such intimate nervous relations, the brain, the stomach, and the liver, are the principal.

But the heart exerts a different and much more powerful influence over the different parts of the body in the æreated blood which it sends into them, and which is indispensable to their life, growth, and active functions. If it performs this duty improperly, disorder of the functions is inevitable; this disturbance is, in general, greater than that effected through the nervous system; but, in varying and undefinable proportions, they are both necessarily present in every case, and often so united that we cannot estimate their respective morbid agencies.

We shall enumerate the principal of these secondary disorders.

1. In the brain and its meninges, we have depression of spirits, pain, vertigo, apoplexy and palsy. Thus, in ossifications of the tricuspid valves, and in passive aneurism of the right ventricle, the torrent in the descending cava, not being received by the heart in due time, the brain becomes engorged, and the maladies which we have enumerated may supervene. We sometime since saw a case of palsy of the parts supplied by the trifacial nerves of one side of the face, that was manifestly owing to this cause. Again. When the left ventricle is hypertrophied, and especially if dilated at the same time, the blood is driven with such impulse into the brain as to disturb its functions, excite pain and inquietude, render the individual unhappy, and finally kill him with apoplexy, or disable him with paralysis. How far the nervous connexion between the heart and brain may contribute to these effects we cannot say, but while a predisposing influence may be charged upon that connexion, the principal blame must rest upon the undue violence of action in the left ventricle.

We cannot doubt that apoplexy and palsy have been much oftener the consequences of diseases of the heart, than has been supposed. Many autopsic inspections after the former maladies, have not been extended to the chest, where the primary læsion might have been detected, and have, therefore, been reported as original affections of the brain.

We suspect, moreover, that inflammations of the encephalon or its membranes, have frequently originated in the disturbed relations of that organ and the heart. In the closing stages of pulmonary consumption, for example, a mild phrenitis is not a very rare occurrence, and sometimes proves the immediate cause of death. This may, in part, be referred to the influence which an inflammatory state of the lungs can exert upon the brain, through the medium of the pneumogastric nerves; but if the læsion of the pulmonary organ has become so great, as to impede the transmission of blood from the right to the left cavities of the heart, it must necessarily accumulate in the former, and by remora, disturb the cerebral circulation. We recollect a case of pneumonic hepatization, which terminated with exquisitely marked phrenitic inflammation, in which the lungs were so impervious, that it was difficult to conceive how they could have in any degree performed the function of transmitting or æreating the blood.

On the whole we might expect, that in many cases of diseased heart, the morbid anatomy of the brain would present extravasations, adhesions, purulent secretions, hydropic effusions, *ramollisement* or softening of the pulpy matter, and various other ravages.

2. The lungs and investing membranes suffer not less than the contents of the cranium. The nervous connexion between the heart and lungs is qui e as intimate as their functional relations. Hence a perverted state of the vital properties of the former, *must* be felt by the latter; and we see it manifested by cough, with or without expectoration, generally the latter, and a spasmodic dyspnœa which frequently resembles asthma. But to the hydraulic connexion of the two organs, we may ascribe a greater number, and those too of a graver character, of the morbid influences which the heart exercises through the respiratory nerves. Thus, if the aortic or left auriculo-ventricular valves, are diseased, the pulmonary veins are unable to empty themselves in due time, and consequently the pulmonary circulation is disturbed. It is, however, the active hypertrophy of the right ventricle, which exerts upon the lungs the most destructive influence, by perpetually injecting its vessels with too much force and volume of blood. Hence arise engorgements, inflammation, hepatization, hæmoptysis, and cellular extravasation or pulmonary apoplexy. The effect of this hypertrophy upon the lungs is precisely of the same kind, indeed, with that of the hypertrophied right ventricle upon the brain. How many diseases of the pulmonary organ have originated from previous disease of the heart, we have not the means of judging; as when both organs are found, on dissection, in a state of disruption, it is not always practicable to assign priority to the disease of either; but we may fairly presume, that a great number of those symptoms which are vaguely denominated asthmatic, are the offspring of maladies of the heart.

3. Next to the lungs, the liver is morbidly affected from cardiac læsions. This influence is no doubt exerted in part through the medium of the nervous system; but still more of the blame is attributable to the right ventricle. When dilatated, with attenuated parieties, it obviously cannot propel forward the blood, with its usual power, and this fluid must, of course, when the circulation is accelerated, accumulate in the auricle and venous trunks. There will then be a struggle between the two cavæ, in which, as gravity lends its aid to the superior, the inferior is liable to be worsted. Hence arise stagnations in the ascending column of venous blood, which, from the proximity of the hepatic veins, are not long in communicating themselves to the liver, and through it to the whole portal system. Thus engorgements of that system, and especially of the liver are established which may eventuate in a phlogistic diathesis, induration, or enlargement. Meanwhile its specific function must be greatly deranged, and hence we find, in advanced states of cardiac maladies, that what are called bilious disorders, are so constantly present, as in some cases to exert an absorbing influence upon the attention of both patient and physician, and may even lead to the opinion, sometimes a correct one, that the disorders in the chest are consequent upon those in the abdomen. This diagnosis, moreover, is rendered plausible, by the beneficial influence which chologogues exert, upon *all* the symptoms under which the patient may be labouring. It is not difficult to understand the mode in which our prescriptions of this class, produce their favourable effects. When a secretory organ is plethoric, the natural cure is increased secretion. In the secondary hepatic disorders now under review, the liver sometimes throws out no bile, at others an excessive quantity, and at others a fluid perverted in its qualities. In the first case by restoring the secretion we diminish the congestion, and in the two latter cases by altering the actions of the liver, and evacuating the excessive secretion from the bowels, we improve the declining health of the abdominal organs, and thus, through the nervous system, ameliorate the sufferings of the heart.

4. The serous and reticulated membranes fall into disease. In active hypertrophy of either ventricle, the capillary system which it supplies, must of necessity be affected by the impulse with which the blood is thrown into it. The disorders thus generated in the brain and lungs we have already considered. A part of the biliary derangement attendant on cardiac diseases, is no doubt owing to the same cause. On the other hand, when the cavities of the heart are passively dilated, the stasis of the blood in the great veins gives rise to a venous plethora, which may be presumed to extend quite to the capillary origins of that system. We have then, in diseases of the hear., both the sanguiferous conditions, which favour serous effusion. To these we may add the lymphatic remora and congestion, which necessarily result from the relations which the great absorbent trunks bear to the venous system. Thus we have too morbid states, which, in different cases, give rise to increased effusion, and another, which tends to diminish absorption. No wonder, then, that dropsical accumulations and infiltrations, present themselves in all parts of the body as secondary affections in diseases of the heart: In short, that we have anasarca, hydropericardium, hydrothorax and ascites. Of these the last is, we believe, the rarest; which may perhaps be ascribed in part, to the great extent of secreting surface which the abdominal viscera enjoy, and through which the engorged capillary system relieves itself. As to the dropsy of the pericardium, its proximate cause is, perhaps, to be found in the turgescence of the right ventricle, which, preventing the return of blood from the substance of the heart by the coronary veins, necessarily renders them plethoric, and thus promotes effusion into the pericardium. When the left ventricle is actively diseased, and the coronary arteries are inordinately injected, effusion may, likewise, be the consequence; and this injection no doubt frequently produces inflammation of the investing membrane, with secretions of pus, or depositions of fibrin and consequent adhesions.

To what extent the serous membranes are affected from sympathy with the heart, we have not the means of judging; but may presume, that a sympathy of that kind exists, especially within the thorax, which may account in part for the greater frequency of dropsical effusions into that cavity than the abdominal.

### Prognosis.

In almost every organic disease, the prognosis must be unfavourable. As yet a method of restraining the the morbid growth or dilation of the heart, retarding the *ramollisement* of its substance, diminishing the deposition of fat, arresting the transformation of its valves into bone or cartilage, or strengthening with new fasciculi of muscle its attenuated parieties, has not been discovered; and until our means of diagnosis shall enable us to detect in their origin, læsions which are only made manifest in their progress we cannot hope to prevent their growth and preserve the parts which they must finally ruin. Hence the great value of the Stethoscope, by which only we can acquire early diagnostic information. But even auscultation is exceedingly limited in the information, which it imparts in the first stages of these (ultimately) mortal derangements. It is some consolation to know, however, that correctly understood and judiciously treated, before they are very far advanced, not a few of them will continue for years or even to the period of old age, without destroying life.

### Treatment.

Organic affections of the heart, as we have already said, are incurable, except they be attacked, in their forming stages, and perhaps even then.

For active hypertrophy and aortic aneurism, the method of Albertini and Valsalva may sometimes do good, if resorted to in due time. It consists in bloodletting and starvation, carried as far as is compatible with the continuance of life. It has lately been said, that this method has done harm in vascular aneurism, as it has been found to reduce the strength of the arterial parieties at a higher ratio than the cardiac, and consequently to increase the power of the latter over the former. But this objection will not lie against the treatment, when applied to hypertrophy of the heart. In this malady the disease consists, essentially, in a morbidly increased action of the coronary arteries; and whatever diminishes the flux of blood into those vessels, must, on theory, retard the morbid growth of the parieties. We may even conceive, that under a course of depletion and abstinence, the heart may become atrophous, like the other parts of the system. Still, upon the restoration of the ordinary quantity of blood, the abnormal actions, reduced but not superseded, may revive; and hence we would suggest, as a second part to the Italian treatment, the administration of digitalis, squills, or colchicum; all of which, experience teaches us, are capable of acting on the vital properties of the heart, in such a manner, as to moderate the force and frequency, and alter the rhythm, of its contractions. This, however, they might do without affecting the nutrient functions of the coronary arteries; and, therefore, the effects of the treatment suggested, might be far short of what these cases require.

Softening of the heart appears to be, generally, the consequence of inflammation, either of its substance or envelopes, and may, therefore, be arrested by the antiphlogistic measures, which cure other inflammations.

As to fatty, osseus and cartilaginous transformations, of the valves or ventricles, we may be said to be entirely ignorant of any method, by which they can be arrested; unless the first might be kept in check, by the means that are found to retard the secretion and deposition of adipose matter, in other parts of the body.

On the whole, the treatment of læsions and degeneracies of the heart, must be palliative—not radical; and we shall close this extended eclectic review, with a brief enumeration of the means which may be employed for this purpose.

I. A careful avoidance of all causes of excitation, internal and external; moral and physical.

II. Abstinence in diet and drinks. Not carried almost to starvation, as in attempting a radical cure; but so regulated as to produce the following effects:

1. A moderate distension of the stomach only.
2. No inordinate stimulation of that organ.
3. No indigestion nor flatulence.
4. A reduced quantity of blood, with a tendency to leanness or atrophy.

The diet should be chiefly milk and vegetable; and the drinks bland and unirritating.

III. Bloodletting. Never to syncope; nor almost to vascular inanition, as when attempting the permanent cure of aortic aneurism; but to the extent necessary to prevent plethora in passive, and reduce the *powers* of the heart in active aneurism. It must be repeated according to the circumstances of each case. It will in general be rendered immediately necessary by the influence of some violent passion, excessive exertion, or inordinate indulgence of appetite, to which the patient may have been subjected.

When from inordinate action of the left ventricle, in active hypertrophy, the brain as we have seen, is either constantly or occasionally injected, and phrenitis, hydrocephalus or apoplexy is likely to supervene, bloodletting is the remedy upon which our chief reliance should be placed.

When, in a similar disease of the right ventricle, the lungs become engorged and inflamed; or their cellular tissue is becoming hepatized; or they are rendered apoplectic, or attacks of hæmoptysis show themselves, bloodletting is equally necessary.

In passive aneurism, or dilatation without hypertrophy, bloodletting is required, not to reduce the powers of the heart, but to carry off the plethora of the venous system. Many affections of the head, up to venous apoplexy, are the offspring of this species of turgesence, and may be relieved by venesection.

The liver and portal viscera suffer from the same repletion of the venous tubes, and will call for the same remedy.

In passive aneurism, moreover, Mr. Burns has shown us, that inflammation of the heart or its membranes, is an occurrence by no means unfrequent, and this also will demand the lancet.

In all cases of passive aneurism, however, we must recollect, that there is great loss of power in the muscular fibres of the heart, which copious bloodletting would augment; while it could not remove the valvular læsions of the left side nor the pulmonary disorganizations which dam up the blood in the cavities of the heart and promote their unnatural distension; and, hence, in this variety of dilatation, and also in the advanced stages of active hypertrophy, our bleedings should, in general, be moderate—not more than 6, 8, 10, or 12 oz. at once.

IV. Cathartics—both chologogue and hydrogogue. These diminish the plethora of the portal circle, and the upper part of the vena cava ascendans, and indirectly reduce the general plethora. They divert from the head, when it is inundated, as well as from the heart. They remove irritating matters from the primæ viæ, which by their impress would, sympathetically, disturb the actions of the heart, and finally they establish in the bowels a centre of irritation and fluxion, which mitigates the wrong and violent action of the parieties of the heart. In the latter stages they may be carried too far. We should then use such as are known most to promote secretion into the bowels—excretion from them is not then so great an object, as in the earlier periods.

V. Diuretics. In active hypertrophy during the first and second stages, sedative diuretics are among the means of subduing the violent actions of the heart, and of lessening the plethora. In these periods, the nitrate of potash, bitartrate of potash, or bitartrate of soda, the squill and the colchicum autumnale are all proper.

In the latter stages of the same maladies, and in passive dilatations, diuretics are still proper: they diminish plethora and divert from the lungs, then exceedingly embarrassed.

It is, however, the dropsical effusions, which so strikingly characterize the final periods of these cardiac dilatations, whether active or passive, that give their chief value to the medicines of this class. Some of these effusions are probably the consequence of the excessive and irregular momentum of the arterial blood—but the greater number are obviously the consequence of venous stagnations. The exhalent and absorbent vessels, moreover, are probably affected, sympathetically, with the heart. These effusions take place into the pericardium, pleura, cellular tissue of the lungs, ventricles of the brain, peritoneum, and lastly the external or subcutaneous parts, and some one of them, or more than one, perhaps, always occurs before death, if the life of the patient should be long protracted.

When the system is vigorous, these dropsical tendencies cannot be corrected without bloodletting; and on the other hand when that remedy is injudiciously or inordinately employed in cardiac læsions, it favours the production of dropsy.

As to diuretics, those just named are proper when there is much vigour of the system; but in reduced and shattered states, the more stimulating should be employed, such as oil of turpentine, cantharides, spirit of nitrous ether, oil of juniper, horse radish, and the tartrate of iron and potash.

VI. Expectorants. These act by carrying off the plethora of the pulmonary vessels. Tartarized antimony, ipecacuanha, squills, colchicum and digitalis are all adapted to this object, when administered under such circumstances as to determine their action upon the lungs. The means necessary to such a determination of their effects are chiefly warm drinks, a well protected state of the surface, and respiring a warm or temperate atmosphere, with the omission of active purgatives.

VII. Counter-Irritants and Revulsants. Mr. Burns recommends a seton over the region of the heart. Blisters to the chest or the extremities might, also, be useful; and both would seem peculiarly indicated in cases of metastasis of rheumatism, gout or cutaneous eruptions, to the heart.

A semicupium or pediluvium often does good, and seems to operate by deriving a great quantity of blood into the lower extremities. Its influence of course is temporary, but might give great relief in violent paroxysms of cardiac and pulmonary embarrassment. In cases of this kind, might not ligatures to the extremities, tight enough to retard the venous blood and cause it to linger in the limbs, be attended with palliation?

VIII. Narcotics and Antispasmodics. Digitalis, opium, colchicum and squills, are observed to diminish the irritability of the heart, and render its contractions slower. In the læsions under consideration, these remedies have all been found beneficial, independently of their effects upon the secretions, and they have done good, it would seem, by abating the excessive sensibility of the diseased organ. It is in consequence of this morbid irritability that the heart is so often in a state of violent, if not powerful, irregular contraction. To this state, if no phlogistic diathesis be present, antispasmodics are adapted, and have been found beneficial. Of these the best are the salts of morphia, assafœdita, sulphuric ether, tinct. of lavender, and ammoniated alcohol.

IX. Tonics and external stimulation. However objectionable tonics may be in most of the stages of hypertrophy and active aneurism, and even in the earlier stages of passive dilatation, towards the close of life when there is great general and cardiac enervation, medicines of this class will contribute to the comfort of the patient. The bitters and chalybeates are then to be administered.

In this stage of these maladies, the circulation is apt to fail in the circumference and extremities, of which coldness, torpor, cellular infiltration and gangrene may be the consequences. In this condition, a hot salt bath, frictions, stimulating liniments, and flannel rollers, may render the patient more comfortable; and postpone the execution of that mandate, from which no human power can grant him a reprieve.

